# Construction and evaluation of a high-density SNP array for the Pacific oyster (*Crassostrea gigas*)

**DOI:** 10.1371/journal.pone.0174007

**Published:** 2017-03-22

**Authors:** Haigang Qi, Kai Song, Chunyan Li, Wei Wang, Busu Li, Li Li, Guofan Zhang

**Affiliations:** 1 Key Laboratory of Experimental Marine Biology, Institute of Oceanology, Chinese Academy of Sciences, Qingdao, China; 2 Laboratory for Marine Biology and Biotechnology, Qingdao National Laboratory for Marine Science and Technology, Qingdao, China; 3 National & Local Joint Engineering Laboratory of Ecological Mariculture, Institute of Oceanology, Chinese Academy of Sciences, Qingdao, China; 4 Laboratory for Marine Fisheries and Aquaculture, Qingdao National Laboratory for Marine Science and Technology, Qingdao, Shandong, China; Xiamen University, CHINA

## Abstract

Single nucleotide polymorphisms (SNPs) are widely used in genetics and genomics research. The Pacific oyster (*Crassostrea gigas*) is an economically and ecologically important marine bivalve, and it possesses one of the highest levels of genomic DNA variation among animal species. Pacific oyster SNPs have been extensively investigated; however, the mechanisms by which these SNPs may be used in a high-throughput, transferable, and economical manner remain to be elucidated. Here, we constructed an oyster 190K SNP array using Affymetrix Axiom genotyping technology. We designed 190,420 SNPs on the chip; these SNPs were selected from 54 million SNPs identified through re-sequencing of 472 Pacific oysters collected in China, Japan, Korea, and Canada. Our genotyping results indicated that 133,984 (70.4%) SNPs were polymorphic and successfully converted on the chip. The SNPs were distributed evenly throughout the oyster genome, located in 3,595 scaffolds with a length of ~509.4 million; the average interval spacing was 4,210 bp. In addition, 111,158 SNPs were distributed in 21,050 coding genes, with an average of 5.3 SNPs per gene. In comparison with genotypes obtained through re-sequencing, ~69% of the converted SNPs had a concordance rate of >0.971; the mean concordance rate was 0.966. Evaluation based on genotypes of full-sib family individuals revealed that the average genotyping accuracy rate was 0.975. Carrying 133 K polymorphic SNPs, our oyster 190K SNP array is the first commercially available high-density SNP chip for mollusks, with the highest throughput. It represents a valuable tool for oyster genome-wide association studies, fine linkage mapping, and population genetics.

## Introduction

Oysters (phylum Mollusca, class Bivalvia) constitute an essential component of many aquatic ecosystems and are economically important in the fisheries and aquaculture industries [1]. Between 2010 and 2014, worldwide production of oysters increased from 4.5 million tonnes to 5.2 million tonnes (FAO 2014; http://www.fao.org), representing the highest global aquaculture production among marine animals. The Pacific cupped oyster *Crassostrea gigas* (Thunberg 1793) originated in northeastern Asia, and is natively distributed along the coasts of China, Japan, and Korea. It was introduced into Europe [2], Australia [3], and America [4] during the 20th century and has since established naturalized populations in most countries where it has been introduced for aquaculture purposes. However, owing to its ability to spread spontaneously and establish itself permanently in new habitats [5, 6], in many regions it is considered as an invasive species. In 2014, production of the Pacific oyster exceeded 625 kilotonnes, with an estimated value of 1.2 billion US dollars (FAO 2014). On the basis of its rapid growth rate, high disease resistance, and adaptability to different environments, the Pacific oyster is one of the most economically important bivalves worldwide.

Sustainable factory farming of oysters requires the development of high-quality oyster strains with rapid growth rates and high disease and stress resistance. Recent studies of the Pacific oyster industry have primarily focused on growth and yield [7–10], summer mortality [11–14], germplasm diversity [15–18], and viral infection [14, 19–21]. In the last decade, studies have mapped important traits [12, 22–24]and whole genome sequencing of *C*. *gigas* has been completed; this facilitates our understanding of the mechanisms of stress adaptation and shell formation in oysters [25]. Nevertheless, despite considerable progress in the oyster industry in recent decades, the Pacific oyster remains at an early stage of domestication, and the molecular mechanisms that modulate the commercially complex traits of this species and help it to survive in the variable marine environment remain unclear.

Single nucleotide polymorphisms (SNPs) are widespread nucleotide variations among individuals of a population, and they constitute the most abundant type of molecular marker in plant and animal genomes. Owing to their high abundance, co-dominant mode of inheritance, and ease of high-throughput detection, SNPs are widely used in biological research [26, 27]. The oyster possesses one of the highest levels of genomic polymorphism among animal species [25], and numbers of SNPs have been identified for various research purposes [28–30]. Nevertheless, oyster SNPs have not been extensively applied in high-resolution genetic research because of the lack of a high-throughput genotyping platform that can simultaneously type thousands of loci in multiple individuals. Such a platform is essential for fine mapping of important traits via extensive linkage or association analysis.

Since the release of the first commercial SNP array by Affymetrix (Santa Clara, CA) in 1996 [31], the use of microarrays and microarray technology has been a feasible choice for large-scale SNPs genotyping. A variety of SNP array platforms have been developed, of which the Affymetrix Custom Array, the Illumina BeadChip (Illumina, San Diego, CA), and the Sequenom MassArray (Sequenom, San Diego, CA) are most popular. These arrays differ in their principles for SNP detection, as well as in their requirements for marker numbers, cost, and sample size. In addition to the human SNP array, SNP arrays have been developed in many animal and plant species, including chicken [32], pig [33], cattle [34], horse [35], catfish [36], common carp [37], Atlantic salmon [38], rainbow trout [39], rice [40], soybean [41], maize [42], and strawberry [43]. In mollusks, a medium-throughput genotyping array involving 384 SNPs has been developed for the Pacific oyster [44]; however, to the best of our knowledge, a high-density oyster SNP array has not previously been available.

Owing to the increasing accessibility of next-generation sequencing (NGS) technologies, genotyping by sequencing (GBS) technologies—which usually detect SNPs through whole or reduced genome sequencing—have become a powerful genetic analysis tool [45]. GBS methods—especially those based on reduced genome sequencing—may be cost-effective for genome-wide SNP discovery or genotyping; however, the disadvantages of GBS arise because NGS data frequently suffer from high error rates derived from multiple factors, including base-calling and alignment errors. In general, for low-coverage sequencing, the larger the number of individuals, the higher the frequency of missing allele calls. For high-coverage sequencing, the increased cost—especially in the case of large genomes—cannot be ignored. When using whole genome sequencing for diploid species, a sequencing depth of more than 15–20 folds is essential for accurate SNP typing [46]. In addition, GBS is dependent on complicated library preparation ensured through rigorous quality control (QC) and intensive subsequent bioinformatics processing steps, including reads cleaning and filtering, reads mapping, brush-fire alignment adjustment, and SNP calling or genotyping; hence, GBS approaches are complex and time-consuming.

Further to the completion of our oyster genome project, we are currently conducting an oyster genome-wide association studies (GWAS) project using a re-sequencing approach to search for genes related to certain complex and important traits. The re-sequencing data generated from wild oysters will provide extensive resources for SNP mining and array design. The aim of the present study was to develop a high-density SNP genotyping array for the Pacific oyster (*C*. *gigas*) based on the Affymetrix Axiom platform, and to assess the potential of this array for future genetic and genomic research.

## Materials and methods

### Ethics statement

The Pacific oyster is a marine bivalve that is broadly distributed in large wild populations in coastal areas. The oysters used in this study were either directly collected from wild populations or cultured by the authors at a local farm. All experiments were performed according to local and national standard regulations. This study was approved by the Animal Care and Use Committee of Institute of Oceanology.

### Sample collection and genome re-sequencing

In this study, we sampled 472 wild Pacific oysters—418 from 18 China coastal locations, 15 from Japan, 24 from Korea, and 15 from Canada. Genomic DNA was extracted from mantle tissues using a standard phenol-chloroform method. For each individual, 5 μg of DNA was sheared into fragments of 200–800 bp using the Covaris system (Life Technologies, Carlsbad, CA). DNA fragments were then treated according to the Illumina DNA sample preparation protocol. Fragments were end-repaired, A-tailed, ligated to paired-end adaptors, and PCR amplified with 300–500 bp inserts for library construction. Sequencing was performed to generate 100-bp paired-end reads with coverage of ≥20 folds on the HiSeq 2000 platform (Illumina) according to the manufacturer’s standard protocols.

### SNP identification

Filtered reads from all individuals were aligned to the oyster reference genome using Burrows-Wheeler Aligner (BWA) software [47] with the parameter “mem -M -t 10 -T 20.” The aligned bam files were sorted and indexed with PICARD tools (https://broadinstitute.github.io/picard/). The reads from SNPs around the insertions and deletions (INDELs) in the bam files were then realigned using the Genome Analysis Toolkit (GATK) module RealignerTargetCreator and IndelRealigner [48]. To obtain high-quality variants, the GATK HaplotypeCaller module and Samtools [49] were used for variants calling of each sample, only concordance variants were selected, the SNPs were filtered with the parameter “QD < 2.0 & FS > 30.0 & MQ < 40.0 & DP < 6 & DP > 888 & ReadPosRankSum < −8.0 & BaseQRankSum < −8”, and the INDELs were filtered with the parameter “QD < 2.0 & FS > 30.0 & ReadPosRankSum < −8.0”. These variants were used to perform Base Quality Score Recalibration (BQSR), and reads were printed for population variants calling. Population variants calling was processed using the GATK HaplotypeCaller module with the parameter “-genotyping_mode DISCOVERY -stand_emit_conf 10 -stand_call_conf 30”.

### SNP selection

Candidate SNPs were filtered in multiple steps using several criteria to eliminate possible false positive sites and distribute SNPs relatively evenly across the genome. For each oyster, if a SNP was of low and excessive read coverage (DP < 10 or DP > 100) and low genotype quality (GQ < 20), the SNP genotype call of the individual was considered to be missing or invalid. For a SNP, the missing rate was defined as the proportion of individuals with a missing SNP genotype in all individuals. The minor allele frequency (MAF) was calculated based on the allele information of individuals without a missing SNP genotype. In this step, SNPs within 20 bp around a predicted INDEL or with MAF < 0.05 or with a missing rate of > 0.1 were filtered out. The remaining SNPs, together with SNPs that were significantly related to important traits such as glycogen, amino acid, fatty acid, and heavy metals contents from our GWAS project (unpublished data), were submitted to Affymetrix for design score assessment using the Axiom myDesign GW bioinformatics pipeline. For each SNP, the sequence feather, binding energies, expected degree of nonspecific binding, possibility of hybridization to multiple genomic regions, and impacts originated from adjacent SNPs were taken into account; a p-convert value—which denotes the probability of the SNP converting to a reliable SNP assay on the Axiom array system—was assigned to each of the two probes flanking an SNP. SNPs with probes having high p-convert values were more likely to be convertible. In addition, a design proposal categorized as “recommended”, “neutral”, “not recommended” and “not possible” was assigned to each of the two probes. The SNP with at least a “recommended” or “neutral” proposal was retained and annotated with in-house custom Perl scripts. The GWAS SNPs and a small number of “large-effect” SNPs—which are predicted to cause stop or start codon loss, introduce a new stop codon, or cause a splice acceptor or donor variant—were initially selected and added into the final array SNP set. For the next selection, every SNP was assigned a priority level value, i.e., 4 for non-synonymous coding, 3 for synonymous coding, 2 for 2-kb upstream/downstream of a gene or in the intron region, and 1 for the intergenic region. The planned SNP capacity was ~200 K, and the assembled oyster genome size was 560 million; hence, the calculated average SNP interval was ~2,800 bp. A 3-kb sliding window was used to scan the oyster genome, and the SNP with the highest priority level value in the window was selected. Next, for genome scaffolds having an observed array SNP number lower than the expected SNP number, a 1-kb sliding window was used to fill in the remaining array probe space. The final list of SNPs was submitted to Affymetrix for production of the Axiom genotyping array.

### Evaluation of the SNP array

To assess the performance of the oyster SNP array, we genotyped 96 Pacific oysters. Among these oysters, 44 were randomly selected from those re-sequenced for SNP discovery. Each oyster was deeply sequenced; hence, the genotype obtained using the oyster SNP array could be compared and cross-checked with that obtained through the GBS approach. We included 24 off-springs of a full-sib family designed for linkage mapping; both parents of this full-sib family were among the 44 randomly selected oysters mentioned above. In addition, 28 oysters were collected from a wild population in Qingdao, China. Genomic DNA was extracted from mantle tissues using a standard phenol-chloroform method. Genomic DNA samples were placed in a 96-well microtiter plate, and adjusted to a final concentration of ~50 ng/μL with a final volume of 10 μL. Genotyping with the oyster SNP chip was conducted using the Affymetrix GeneTitan Multi-Channel (MC) Instrument according to the standard operating instructions. The raw data stored in the form of CEL files were imported into Affymetrix Axiom Analysis Suite software version 1.1.1.66 for QC analysis and genotype calling, using the Axiom GT1 cluster algorithm with default parameters. The genotyping results were processed using SNPolisher software version 1.5.2. To assess the usability of the array in population genetics, all samples were subjected to principal component analysis (PCA) using Eigensoft software [50].

## Results and discussion

### Discovery and selection of high-confidence SNPs for array construction

**Fig 1:**
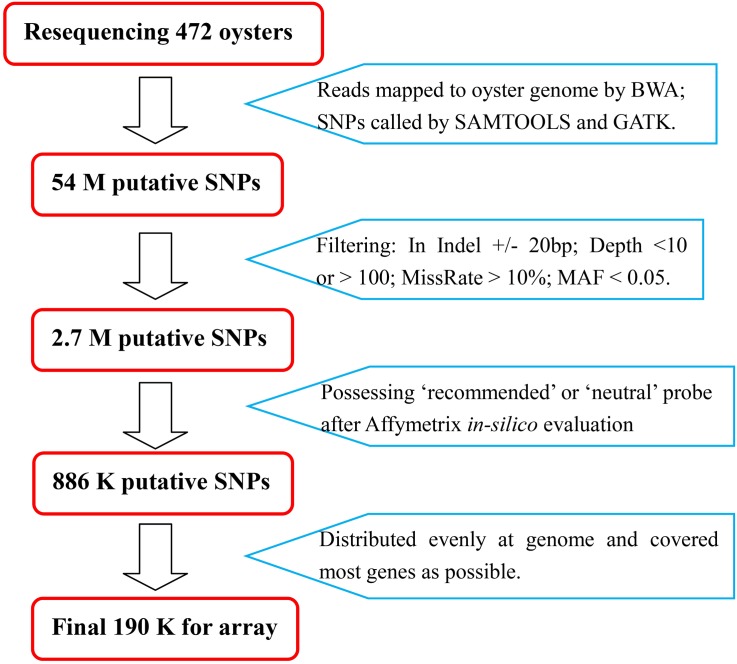
Flow diagram for the SNP selection steps with major criteria.

**Fig 2:**
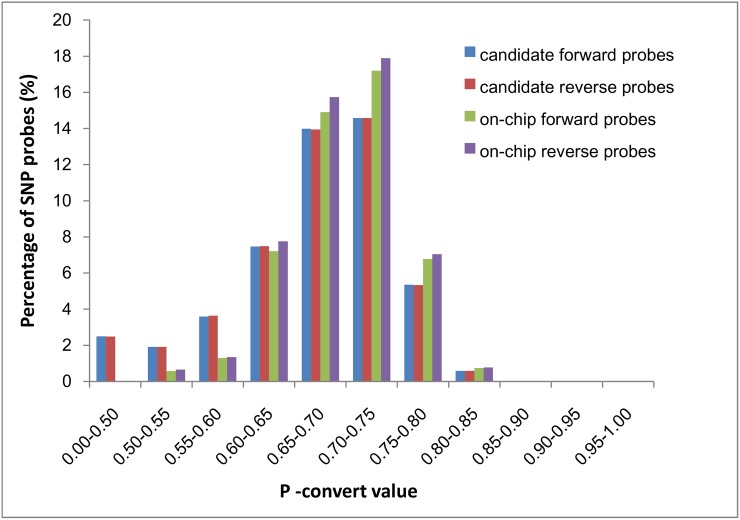
Distribution of the p-convert values for candidate and on-chip probes.

For SNP discovery, we re-sequenced 472 wild oysters with coverage of >20 folds for each individual. After aligning the reads to oyster genome reference sequences through BWA, we identified 54 million high-quality SNPs using Samtools and GATK software; these SNPs may constitute the largest available oyster SNP dataset with broad representation of oyster resources. By applying several filters (see Materials and Methods), we reduced the initial SNP number by a factor of 20, to ~2.7 million putative SNPs. After *in silico* analysis for reproducibility prediction by Affymetrix, 886 K SNPs passed the evaluation; based on the distribution and function of each SNP, we submitted a final list of 190,420 SNPs to Affymetrix for production of an oyster genotyping array (Fig 1, S1 Dataset). We synthesized 192,789 probes for the 190,420 SNPs on the chip; 188,051 SNPs were tiled with one set of probes, and the remaining 2,369 SNPs were tiled with two sets of probes. For the 886 K candidate SNPs categorized by Affymetrix as “recommended” or “neutral”, approximately 84% of the p-convert values of probes were >0.60. Probes with higher p-convert values are more likely to be converted; hence, 96% of the p-convert values of the 192,789 probes for the 190 K on-chip SNPs were >0.60 (Fig 2).

### Genotyping performance of the SNP array

We performed a series of QC steps to ensure the accuracy of the outcomes during genotype calling. The signals of all 96 samples possessed a dish QC (DQC) value of 0.92–1.0, which was much greater than the default Axiom DQC cutoff value of 0.82. However, one wild oyster sample did not pass the call rate (CR) QC because its CR was 0.954, which was slightly lower than the default cutoff value of 0.970; hence, the overall rate of passing samples was 0.99 (95/96). As predicted, the passing rates of the 44 re-sequencing samples and the 24 full-sib family samples were 1.0, and there was no significant difference between the passing rate of the 28 wild samples (27/28 = 0.96) and that of the 44 re-sequencing samples (Fisher’s Exact Test, *P* = 0.39). All the raw data stored in CEL format have been deposited in the NCBI GEO database with an accession number of GSE94633.

The genotypes of the oyster SNPs were classified into the following six categories according to their clustering performance (S1 Fig, S2 Dataset): (i) “PolyHighResolution”—three clusters were formed with good resolution; (ii) “NoMinorHom”—two clusters were observed with no samples of one homozygous genotype; (iii) “MonoHighResolution”—a single cluster of a homozygous genotype was formed; (iv) “OTV,” off-target variants—three good clusters were formed, with a single additional off-target cluster caused by variants in the SNP flanking region; (v) “CallRateBelowThreshold”—the SNP call rate was below the threshold (0.970), but other cluster properties were above the threshold; and (vi) “Other”—the SNPs were not grouped into any of the previous categories. We found that 101,193 SNPs (representing 53.1% of the total on-chip SNPs) were polymorphic, with three observed SNP genotypes (Table 1). Together with the 32,791 “NoMinorHom” SNPs, 133,984 (~70%) of the SNPs were validated as polymorphic and successfully converted on the chip.

**Table 1:** The counts of the SNPs clustering categories.

SNP Category	Probe No.	Percent	SNP No.	Percent
PolyHighResolution	101,722	52.8	101,193	53.1
NoMinorHom	33,033	17.1	32,791	17.2
MonoHighResolution	4,177	2.2	4,145	2.2
OTV	3,472	1.8	3,409	1.8
CallRateBelowThreshold	17,336	9.0	17,015	8.9
Other	33,049	17.1	31,867	16.7
Total	192,789	100.0	190,420	100.0

### Statistical analysis of the converted SNPs

**Fig 3:**
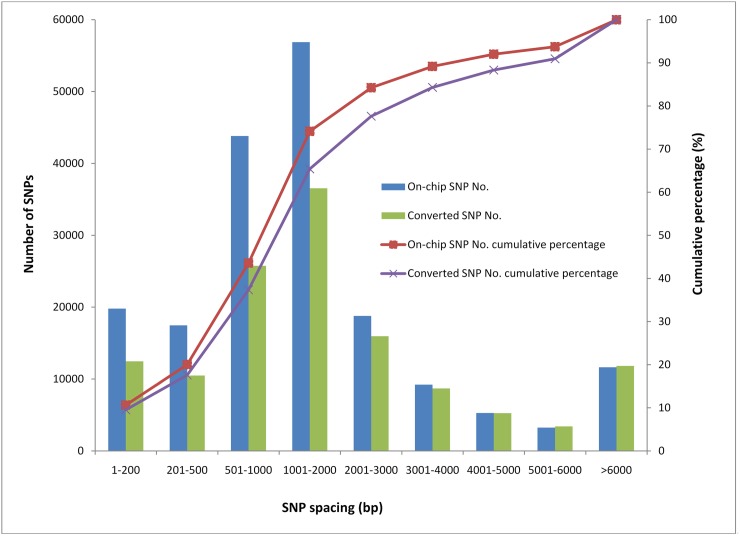
Distribution of the interval spacing of the SNPs on the array.

Transition SNPs comprised 73% of the polymorphic SNPs, including 49,108 A/G and 48,442 C/T; transversion SNPs involved 14,863 A/C, 15,047 G/T, 4,437 A/T, and 2,087 C/G (Table 2). The average conversion rate was 0.70, and the conversion rate of each type of SNP was between 0.66 and 0.79. According to the positions of the SNPs in the genome or their effects on the predicted protein sequences, ~44.4% (59,485) of the converted SNPs were in the coding region—these SNPs included 39,579 synonymous SNPs and 19,906 non-synonymous SNPs (S3 Dataset). The SNPs in the intron region, 2-kb upstream/downstream of a gene, and in the intergenic region accounted for 22.5%, 16.1%, and 17.0% of the converted SNPs, respectively (Table 3). Thus, 111,158 (83%) SNPs were in or near a predicted coding gene of the oyster genome and they may facilitate future gene-related analysis of oysters. The on-chip SNPs were located in 4,315 genome scaffolds that spanned a total length of 539.7 million; the average interval spacing of the SNPs was 2,960 bp. The converted SNPs were located in 3,595 scaffolds with a length of ~509.4 million; the average interval spacing was 4,210 bp. To assess the evenness of the SNPs, we calculated the distribution of the converted SNPs. We found that 12,458 (9.6%) SNPs had a small inter-SNP spacing (<200 bp) and 10,487 (8.0%) SNPs had a small SNP spacing (200–500 bp) (Fig 3). In addition, 25,756 (19.8%), 36,547 (28%), 15,959 (12.2%), 8,701 (6.7%), and 5,246 (4.0%) SNPs had a medium marker spacing of 500–1000 bp, 1000–2000 bp, 2000–3000 bp, 3000–4000 bp, and 4000–5000 bp, respectively. Cumulatively, 37.4% SNPs had a marker spacing of <1000 bp, 51.0% SNPs had a marker spacing of 1001–5000 bp, and 11.6% SNPs had a marker spacing of >5000 bp. Moreover, the 111,158 SNPs were distributed in 21,050 coding genes, with an average of 5.3 SNPs per gene. Of the genes, 2,750 (13.1%) had only one SNP converted on the chip, 11,460 (54.4%) had 2–5 SNPs converted on the chip, 4,561 (21.7%) had 6–10 SNPs converted on the chip, and 2,279 (10.8%) had more >10 SNPs converted on the chip.

**Table 2:** The counts of the SNPs types.

SNP type	On-chip	Percent	Converted	Percent	Conversion Rate
A/G	68,655	36.1	49,108	36.7	0.72
C/T	67,800	35.6	48,442	36.2	0.71
G/T	22,345	11.7	15,047	11.2	0.67
A/C	22,241	11.7	14,863	11.1	0.67
A/T	6,753	3.5	4,437	3.3	0.66
C/G	2,626	1.4	2,087	1.6	0.79
Total	190,420	100	133,984	100	0.70

**Table 3:** Summary of the SNPs according to their positions or functions.

SNP region/function	On-chip	Converted
Coding region		
**synonymous**	46,285	39,579
**missense**	24,215	19,604
**stop_gained**	245	186
**start_lost**	57	41
**stop_lost**	45	30
**stop_retained**	45	40
**initiator_codon**	6	5
Intron region		
**intron**	43,304	27,790
**splice_donor**	83	61
**splice_acceptor**	63	45
**splice_region**	2,745	2,259
2Kbp-up/down-stream of a gene		
**up**	17,630	11,880
**down**	15,133	9,638
Intergenic region	40,564	22,826

### Concordance of the SNP genotypes between array and re-sequencing

**Fig 4:**
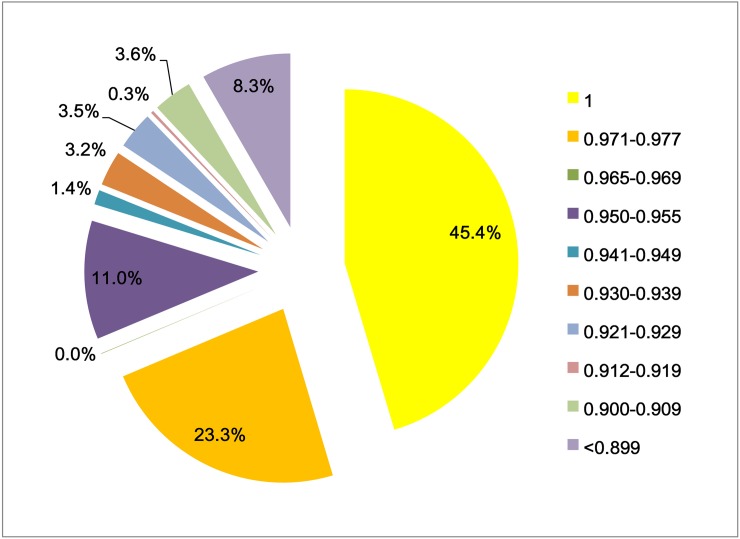
Distribution of the concordance rate of the SNPs on the array.

To further evaluate the quality of the converted SNPs, we compared the genotypes obtained from the SNP array with those obtained through high-depth re-sequencing. We found that 60,805 SNPs (representing 45.4% of the total on-chip SNPs) had a concordance rate of 1.000, 31,181 SNPs (representing 23.3% of the total on-chip SNPs) had a concordance rate of 0.971–0.977, and 14,777 SNPs (representing 11.0% of the total on-chip SNPs) had a concordance rate of 0.950–0.955 (Fig 4). Cumulatively, 68.7% of the converted SNPs had a concordance rate of >0.971, and the mean concordance rates for the converted SNPs and all the on-chip SNPs were 0.966 and 0.927, respectively. The non-concordance rate (~0.034) implied the presence of errors either in the array or in the GBS-derived genotypes; however, the data showed that the oyster SNP array and the GBS technology were both appropriate for oyster SNP genotyping and provided high-quality genotyping outcomes with an average accuracy rate of >0.96.

### Evaluation of the genotyping accuracy according to family data

We further evaluated the genotyping accuracy using data obtained from the two parents and 24 off-springs of a full-sib family. The numbers of genotype combinations of the two parents that could be used in linkage analysis for types “AA × AB”, “AB × AA” and “AB × AB” were 20,716, 20,814, and 10,271, respectively (Table 4). No errors were detected in the markers of type “AB × AB” and the error rate for types “AA × AB” and “AB × AA” was ~0.025. In the case of type “AA × BB”, all off-spring genotypes were expected to be “AB” heterozygosity; however, some homozygous individuals were detected and thus a very high genotype error rate was observed in markers of type “AA × BB”. We detected 9,857 SNPs with unexpected genotypes; these SNPs accounted for 7.4% of the 133,889 SNPs that could be typed in both parents. For each individual, the number of unexpected genotypes ranged from to 3,228 to 3,445, and the average accuracy rate was 0.975. The average CR and concordance rate of the 9,857 SNPs were lower than the mean levels, indicating that these SNPs could not be well typed and that their genotyping results must be treated with caution in subsequent analysis. The oyster genome is highly polymorphic; hence, we deduced that these SNPs may be artificial and that the genotyping errors may result from the unspecific binding of probes.

**Table 4:** Estimation of genotyping accuracy by family data.

Parents genotypes	All no.	Error no.	Error no. Percentage	Call rate	Concordance rate	Error rate
AA × AA	73,246	2,040	2.8	0.996	0.971	0.002
AA × AB	20,716	2,199	10.6	0.995	0.964	0.025
AA × BB	8,842	3,342	37.8	0.994	0.943	0.235
AB × AA	20,814	2,276	10.9	0.995	0.962	0.026
AB × AB	10,271	0	0.0	0.995	0.962	0.000
	133,889	9,857	7.4	0.995	0.966	0.025

### Population structure analysis

**Fig 5:**
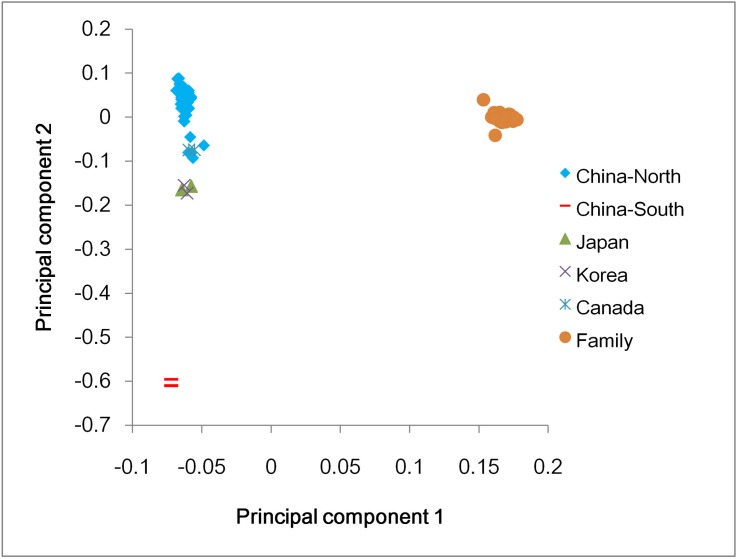
Principal component analysis of all samples. The first principal component (PC1) was assigned to X axis, and the second principal component (PC2) was assigned to Y axis. “China-North”, “China-South”, “Japan”, “Korea”, and “Canada” represented the Pacific oysters collected in northern China, southern China, Japan, Korea, and Canada, respectively. “Family” represented the parents and 24 off-springs of a full-sib family. The parents of the full-sib family were also collected in northern China.

The overall genome similarity of any two samples and the population stratification has frequently been estimated in population genetics and GWAS analyses [51]. Based on the genotypes of the 133,984 converted SNPs, the genetic differentiation was directly observed and the samples were divided into three groups according to the first and second principle component (Fig 5). Individuals in the same family clustered together and were always distinct from others. Pacific oysters collected in southern China, which were identified as subspecies of *C*. *gigas* [52], clearly differed from Pacific oysters collected in northern China, Japan, Korea, and Canada. The largest group consisted of Pacific oysters collected in northern China, Canada, Korea, and Japan; moreover, the samples collected in northern China could be further distinguished from those collected in Korea and Japan (Fig 5, S5 Dataset). The sample size in our present study was not large enough for comprehensive research; nevertheless, our results were in concordance with our expectations and may, to some extent, provide an insight into the population genetics of Pacific oysters.

## Conclusions

In this study, we have established the largest commercially available Pacific oyster SNP array, composed of 190 K SNPs. Genotyping of 96 oysters revealed that ~133 K SNPs (~70%) were polymorphic and successfully converted on the chip. The SNPs were distributed evenly throughout the oyster genome, located in 3,595 scaffolds; the average interval spacing was 4,210 bp. In addition, 111,158 SNPs were distributed in 21,050 coding genes, with an average of 5.3 SNPs per gene. Comparison of the array-derived genotypes with those obtained through re-sequencing revealed that the mean concordance rate was 0.966. Moreover, evaluation based on the genotypes of two parents and 24 off-springs of a full-sib family revealed that the average accuracy rate was 0.975. Our results indicate that the oyster SNP array constitutes an alternative platform for genome-wide SNP genotyping and represents a valuable tool for research into the genetics of *C*. *gigas*.

## Supporting information

S1 Dataset The sequences of all the on-chip SNPs. (GZ)

S2 Dataset The clustering category for each SNP probe. (GZ)

S3 Dataset The function annotation of all the on-chip SNPs. (GZ)

S4 Dataset The SNP genotypes for the 96 samples. (GZ)

S5 Dataset The PCA data of the 96 samples. (GZ)

S1 Fig Examples of the six SNP cluster categories. SNPs could be classified into six categories according to the probeset clustering properties: (i) ‘PolyHighResolution’; (ii) ‘NoMinorHom’; (iii) ‘MonoHighResolution’; (iv) ‘OTV’; (v) ‘CallRateBelowThreshold’; and (vi) ‘Other’.
(TIF)
