# Supplementary figures and images for: Construction and evaluation of a high-density SNP array for the Pacific oyster (Crassostrea gigas)
Source: PLoS One. 2017 Mar 22;12(3):e0174007. doi: 10.1371/journal.pone.0174007 (PMC5362100; doi:10.1371/journal.pone.0174007)

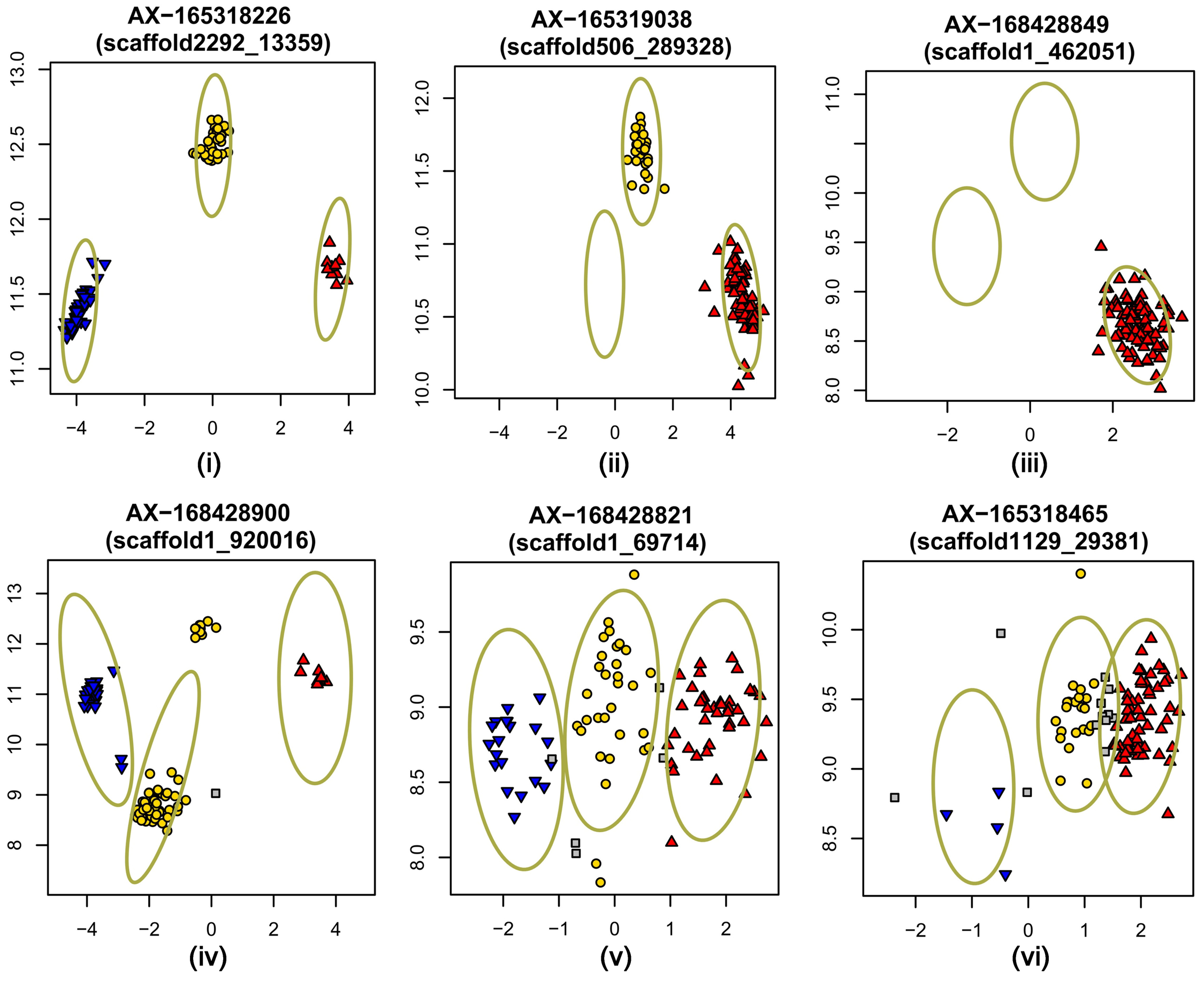

Supplement: S1 Fig — SNPs could be classified into six categories according to the probeset clustering properties: (i) ‘PolyHighResolution’; (ii) ‘NoMinorHom’; (iii) ‘MonoHighResolution’; (iv) ‘OTV’; (v) ‘CallRateBelowThreshold’; and (vi) ‘Other’. (TIF) [file pone.0174007.s006.tif]
